# Exploring longitudinal physiologic stress measurement and sleep quality interventions to improve psychological well-being in nurses: a pilot study

**DOI:** 10.1080/21642850.2025.2503376

**Published:** 2025-05-13

**Authors:** Allison A. Norful, Krystyna de Jacq, Jiawen Zhao, Yuandi Gao, Kathryn Asadoorian, Yilei Yang, Hyun Jin Jung, Ari Shechter

**Affiliations:** a School of Nursing, Columbia University, New York, NY, USA; b SUNY Polytechnic Institute, Utica, New York, USA; c Mailman School of Public Health, Columbia University, New York, NY, USA; d New York Presbyterian Hudson Valley Hospital, Cortlandt, NY, USA; e Columbia University Irving Medical Center, New York, NY, USA

**Keywords:** Sleep, nurses, depression, mental health, work environment

## Abstract

**Introduction:**

Rates of depression, burnout, and anxiety among nurses are high. Improving sleep quality may mitigate psychological distress, but research on effective sleep interventions for nurses is limited. This pilot study explored the preliminary effects of the Somni© sleep quality kit on sleep, stress, and psychological health among nurses using continuous physiologic data from the Oura ring©.

**Methods:**

A prospective pilot trial was conducted with 25 nurses. Participants wore the Oura ring© for eight weeks to collect data on heart rate variability (HRV) and sleep. The Somni© sleep kit, containing evidence-based sleep aids, was introduced from weeks 4-8. Participants completed surveys at baseline, 4, and 8 weeks to assess sleep quality, stress, burnout, and depressive symptoms. Data were analyzed using paired t-tests and linear mixed-effect models.

**Results:**

The sample was predominantly White (64%), non-Hispanic (88%), and female (84%). Lavender spray and white noise machines were the most frequently used sleep aids. Self-reported sleep latency significantly decreased (*p* = 0.03), with a trend toward improved sleep quality. No significant changes were observed in physiologic metrics or depressive symptoms. Effect sizes ranged from moderate to small, with the greatest improvement in sleep latency.

**Discussion:**

The Somni© sleep kit showed potential for improving self-reported sleep quality and sleep latency, especially through non-pharmacologic interventions. While the results were mixed, this study supports the feasibility of using wearable devices to track sleep and stress in nurses. Future research should include larger samples and investigate the long-term effects of sleep interventions on nurses’ mental health.

## Introduction

Rates of depression, burnout, suicide, and anxiety among nurses have reached alarming rates. A recent survey conducted across 13 countries found that nurses nurses who reported difficulty sleeping were 15 times more likely to experience symptoms of depression (Norful et al., 2024). There is substantial evidence that improved sleep quality may serve as a protective factor against poor psychological health. However, there is limited knowledge about sleep characteristics among nurses and further, which interventions may improve sleep in nurses. The American Association of Sleep Medicine (AASM) recommends approximately seven hours of sleep every 24 hours (American Academy of Sleep Medicine, 2023). Yet, according to *Diagnostic and Statistical Manual of Mental Disorders-5th edition* (American Psychiatric Association, 2022), about one-third of adults in general populations report some symptoms of sleep disorders, such as insomnia. A sleep-wake cycle is dependent on one’s own circadian rhythm, synchronized with the 24-hour clock. The circadian rhythm is influenced by one’s environmental factors such as daylight and night exposure. In nurses, many of whom are shift workers, there is a possibility of misalignment between their circadian rhythm and the needs of their shift work.

The relationship between insomnia and mental disorders is bidirectional; symptoms of insomnia are included in various mental disorders and continuing insomnia is a risk factor for depression, anxiety, and alcohol use disorders. Lee et al. (2021) conducted a sleep study with 62 nurses working full-time in a hospital setting. The authors reported that majority of the participants (68%) reported insomnia-related issues. Furthermore, almost all participants (95%) stated that they would like to participate in sleep-insomnia intervention, underlining the need for such interventions. Researchers have found promising results testing the impact of insomnia treatment (e.g. cognitive behavioral therapy) among individuals with depression (Hertenstein et al., 2022). Yet, there appears to be a scarcity of evidence surrounding which sleep interventions are preferred by or effective in supporting sleep quality among the nursing workforce. Existing studies are often limited by self-report surveys and cross-sectional design, thus limiting causality. To optimize sleep quality in nurses, and potentially mitigate poor mental health risk, rigorous trials are needed.

In the wide breadth of sleep research, multiple sleep quality promotion devices have been tested and demonstrate a significant impact on overall sleep quality. Interventions such as aromatherapy (Salamung & Elmiyanti, 2023), music therapy, relaxation techniques, and sleep aids have been shown to have medium to large improvements in sleep quality (Beswick et al., 2023). In contrast, there is variable evidence about the impact of other sleep-promoting interventions such as blue light-blocking glasses or earplugs (Yeretsian et al., 2023). It remains unclear which interventions nurses view as effective and whether they are impacted or accepting of such sleep quality promotion interventions.

In this present study, we aimed to pilot the use of an industry-developed sleep quality promotion kit, called *Somni^©^*, which includes evidence-based sleep promotion devices (e.g. eye masks, aromatherapy, blue light-blocking glasses). Since sleep intervention preferences are often individualized and the effectiveness of such interventions among nursing workforce remains unknown, we chose to explore a kit with multiple sleep promotion devices as opposed a tailored intervention. To avoid study bias stemming from self-report and cross-sectional approaches, we included physiological and behavioral measures to capture real time sleep data. The overall purpose of this study was to longitudinally explore the preliminary impact of an existing sleep quality promotion intervention (*Somni^©^*) on physiologic stress (heart rate variability), sleep health (self-reported sleep quality, daily sleep duration, REM sleep duration), and psychiatric health outcomes including depression and burnout among nurses. More specifically this pilot study aimed to: (1) test the feasibility and acceptability of using Oura rings among nursing workforce participants; (2) obtain preliminary participant preferences for sleep intervention devices; and 3) understand preliminary associations between sleep quality, physiological data (HRV and sleep duration, sleep latency, time spent in REM cycle) and mental health outcomes. We hypothesized that participants would improve sleep duration, and have reduced depression, burnout, perceived stress, and anxiety scores following the implementation of the So*mni^©^* sleep kit. Pilot data stemming from this study will be used to understand nurse preferences for sleep quality promotion interventions and the subsequent perceived impact on sleep quality. The data will also inform a fully powered trial with tailored interventions to investigate occupational factors associated with stress or suboptimal mental health, and the impact of evidence-based sleep interventions in nurses.

## Materials and methods

### Design, sample, and data collection

This study used a prospective pragmatic pilot trial design. Ethical approval was obtained by Columbia University Irving Medical Center institutional review board (Protocol number: AAAU3204). We recruited a convenience sample of 25 nurses from a community-based teaching hospital in close proximity to the study team. A hospital wide email was sent to all clinical nurses explaining the study, its purpose, study procedures and contact information for the study team. The first 25 respondents that expressed interest met eligibility criteria were enrolled (funding was limited sample to a maximum of 25 participants). Sample sizes ranging from 10 to 40 have been deemed appropriate in providing estimates precise enough to inform a power analysis and generate sufficient preliminary data (Hertzog, 2008). Participants were eligible if they were: (1) currently practicing as a registered nurse in the clinical setting; (2) have worked continuously in the same position for at least 1 year; and (3) read, speak, and understand English language. Participants were excluded if (1) > 1-month sick leave in the past 3 months; (2) pregnancy (known physiologic stress and sleep confounder (Bleker et al., 2017)); and (3) other healthcare workers (e.g. physicians). A recruitment email was distributed by nursing leadership hospital-wide to all clinical nurses. Written informed consent was obtained by the principal investigator from all study participants prior to the start of the study. At the start of the study, each participant was assigned a unique ID number that was used to track study procedures and enhance anonymity. Only the PI and research assistant had access to the identity of participants, to follow up if needed with email reminders for each phase of the study.

### Physiologic biomarkers (sleep data and heart rate variability)

Participants wore a device *Oura ring^©^*, on their finger 24-hours per day for eight weeks to collect continuous measures of heart rate and daily sleep. The *Oura ring^©^* is a piece of jewelry that can be custom fit to each participant, collects continuous physiologic measures, and syncs to the participants phone via an app. Researchers have noted that the Oura ring demonstrates accuracy for total sleep time comparable to research-grade actigraphy with low bias for capturing REM sleep (Kainec et al., 2024). The data synched from the participant’s phone is then visible on a web-based dashboard for the study team to extract. During the study the participants were not able to see the dashboard, so they did not get real-time feedback, as this could impact findings. The device collected sleep data including daily sleep onset latency (time to fall asleep), total sleep time, time spent in REM sleep. We also measured heart rate variability (HRV), a highly sensitive physiologic metric for stress. HRV is the constant variation in milliseconds between beats and controlled by the autonomic nervous system. Low HRV is a strong indicator of physiologic stress (fight or flight response) and has been associated with suboptimal mental health, such as increased risk for suicide attempts (Wilson et al., 2016). Studies have demonstrated high validity in the assessment of HRV and sleep using the *Oura ring^©^* dashboard (Kinnunen et al., 2020; Malakhatka et al., 2021). Specifically, researchers have found that the Oura ring dashboard’s heart rate (HR) and root mean square of successive differences (RMSSD) showed low error variance compared to HRV parameters from normal interbeat intervals, with strong positive correlations (*P* < .001) for HR, RMSSD, average of normal heart beat intervals (AVNN), and percentage of successive normal beat-to-beat intervals that differ by more than 50 ms (pNN50), while SD of normal beat-to-beat intervals (SDNN) and high frequency (HF) had moderate correlations and low frequency (LF) and LF:HF ratio had weak correlations. The average-per-night test demonstrated high positive relationships (*P* < .001) for most parameters, moderate for LF:HF, and lower error variance than a 5-minute test, with narrower 95% CIs for HR, RMSSD, AVNN, and pNN50 but wider CIs for SDNN, LF, HF, and LF:HF ratio (Cao et al., 2022).

### Sleep intervention

In a run-in period, during the first 4 weeks of data collection, participants were instructed to perform their routine habits related to work and sleep while wearing the Oura ring. Then, from Week 4 thru 8, we instructed the participants to use the sleep-aid devices in the So*mni^©^* sleep kit. *Somni^©^* is an evidence-based personalized sleep intervention aimed at promoting optimal sleep quality and has demonstrated effectiveness in shift workers outside the healthcare sector (e.g. Disney) (Berg, 2022). The sleep kit is a box of items with demonstrated past evidence of improving sleep quality: Blue light blocking glasses (Hester et al., 2021), white noise machine (Riedy et al., 2021), lavender spray (Fismer & Pilkington, 2012), ear plugs (Jones & Dawson, 2012), light blocking eye mask (Lopez & Boncyk, 2021), and herbal tea (Baek et al., 2018). Rather than dictate a ‘one size fits all’ approach to optimize sleep quality, there is current evidence that individualized sleep interventions are recommended to optimize sleep quality (Ye & Dykes, 2021). *Somni^©^* promotes individualized sleep quality promotion and participants are able to select their preferred use, timing, and duration of each of the box contents. In this pilot phase we aimed to understand individual preferences for each of the devices and therefore instructed participants to select and report their individual use of each device daily, as opposed to tailoring a specific schedule of device uses.

### Electronic quantitative surveys

We administered electronic surveys via Qualtrics survey software (Qualtrics, 2022) at baseline, 4 and 8 weeks to align with the intervention application. The first 4 weeks there was no intervention. During weeks 5 through 8, the participants were instructed to implement the intervention. The survey consisted of demographic questions and validated measures for personal characteristics, sleep, stressful life events, psychiatric characteristics, work environment factors, and psychological health outcomes. All measures had established psychometric validity and reliability and have been tested by the study team in previous studies. The week 8 survey also consisted of additional single items to assess the frequency of use for each individual Somni© sleep kit aid, perceived impact on quality of sleep, and whether the individual used a pharmaceutical-based sleep pharmacologic agent (e.g. Ambien, melatonin).

**Burnout –** The Maslach Burnout Inventory (Maslach & Jackson, 1981) (MBI) was used as the measure of job burnout. The MBI consists of 3 subscales: emotional exhaustion (9 items) (α = 0.8); depersonalization (5 items) (α = 0.79) and professional accomplishment (8 items) (α = 0.82). Each subscale has a 7-point Likert-type response scale ranging from ‘never’ (0) to ‘every day’ (6). A composite score is calculated for each subscale individually. The higher the scores on emotional exhaustion and depersonalization subscale, the greater the job burnout. The lower the scores on the personal accomplishment subscale, the greater the job burnout.

**Stress** – The Perceived Stress Scale (Cohen, 1988) is a 10 item instrument that asks participants to rate the frequency of their stress in the past month. Response options include a 5-point Likert-type scale from ‘never’ (0) to ‘very often’ (4). The higher the total score the greater the level of stress. Scores less than 13 indicate low stress while scores greater than 27 indicate high stress.

**Depressed mood** – The Beck’s Depression Inventory-II (BDI-II) (Beck et al., 1996) is a 21 item instrument that assesses respondents’ presence and frequency of depressive symptoms according to Diagnostic and Statistical Manual for Mental Disorders criteria. Response options include Likert-type scale ranging from 0 (absence of the symptom) to 3 (strongest endorsement of the symptom). The higher the total score the greater probability of depression.

**Pittsburgh Sleep Quality Index (PSQI)** (α =  0.68 to 0.84) consists of 19 self-rated questions and evaluates seven components: (1) Subjective sleep quality – individual's perception of their sleep quality; (2) Sleep latency – time taken to fall asleep; (3) Sleep duration – total hours of sleep per night; (4) Habitual sleep efficiency – ratio of total sleep time to time spent in bed; (5) Sleep disturbances – frequency of issues like waking up at night; (6) Use of sleep medication – frequency of sleep aid usage; and (7) Daytime dysfunction – impact of sleep issues on daily activities. Each component is scored from 0 to 3, with a total PSQI score ranging from 0 to 21. A score greater than 5 indicates poor sleep quality, while lower scores suggest good sleep quality.

### Data analysis

All data was merged into a single Excel spreadsheet, cleaned and coded, and then exported to R software for analysis. First, we calculated composite scores of individual measures (sleep, stressful life events, psychiatric characteristics, and work environment characteristics). Descriptive statistics were calculated including means and standard deviations (normally distributed values), median and interquartile range (non-normally distributed variables), or absolute and relative frequencies (categorical variables). The significance level was set at 0.05. We tused t-tests to determine differences before and after the intervention at a one month follow up interval. Then we used a linear mixed effect model to determine differences before and after the intervention. Models were adjusted for age, race, gender, age, and ethnicity based on existing evidence about variation and differences in heart rate variability across biologic characteristics (Garavaglia et al., 2021). Finally, given the pilot sample size, we calculated Cohen’s d to determine effect sizes.

## Results

A total of 25 participants voluntarily enrolled in the survey. At week 7 of 8, 1 participant lost the Oura ring and therefore stopped physiologic data collection – they did complete the final survey. We otherwise had full compliance with all participants wearing the Oura ring 24 hours a day for the full 8 weeks. The sample was predominantly White (64%), non-Hispanic (88%), and female (84%) (Table 1). The mean age of participants was 45 years old and reported an average of 18 years of clinical experience, while the average number of years in their current position was 4 years.

**Table 1.** Participant demographics and work characteristics.

	Overall
**Demographic/Characteristic**	**(*N*** **=** **25)**
**Age**	
Mean (SD)	45.2 (11.6)
Median [Min, Max]	44.0 [24.0, 72.0]
**Years of clinical experience**	
Mean (SD)	18.0 (12.5)
Median [Min, Max]	17.0 [2.00, 45.0]
	**n (%)**
**Race**	
Asian	3 (12.0%)
Black	2 (8.0%)
Native Indian	1 (4.0%)
Other	3 (12.0%)
White	16 (64.0%)
**Gender**	
Female	21 (84.0%)
Male	4 (16.0%)
**Marital status**	
Divorced	5 (20.0%)
Married	15 (60.0%)
Never married	3 (12.0%)
Partner	2 (8.0%)
**Highest degree**	
Assiciated degree/ certificate in nursing	4 (16.0%)
Bachelor	14 (56.0%)
Master	7 (28.0%)
**Length of commute**	
> 30	12 (48.0%)
11∼20	3 (12.0%)
21∼30	5 (20.0%)
5∼10	5 (20.0%)

Of the devices in the Somni© sleep kit, the Lavender aromatherapy spray and the white noise machine were reported to be used at the highest rates, with 20% (*n* = 5) and 16% (*n* = 4) of participants reporting use at least 5 days per week (Table 2). This was followed by ear plugs and ear masks, each reported to be used by 12% (*n* = 3) of participants at least 5 days per week. No participants reported the use of the nasal strips. Although reported to be used relatively less (8% reporting use at least 5 days per week), almost one-third of participants perceived improvements in sleep quality following the use of the blue-light blocking glasses. The lavender spray was also perceived to improve sleep quality in about a third of participants. Although not included in the sleep kit, 20% of participants reported using personal medications (e.g. Ambien, melatonin) at least 5 days per week, and of those participants, almost one-third reported improved sleep quality as a result.

**Table 2.** Use of Somni© sleep kit components and perceived impact.

Device	Percentage of participants using device at least 5 days/week	Perceived improvement in sleep quality	
	n (%)	n (%)	
White Noise Machine	4 (16)	6 (23)	
Ear Plugs	3 (12)	4 (16)	
Eye Mask	3 (12)	7 (28)	
Herbal tea	1 (4)	6 (24)	
Blue-light blocking glasses 2 (8)		9 (36)	
Nasal Strips	0 (0)	3 (12)	
Lavendar Spray	5 (20)	8 (32)	
Personal Medication or Supplement (e.g. Ambien, Melatonin)^a^			
	5 (20)	8 (32)	

^a^ Not included in sleep kit but reported by participant.

Unadjusted paired t-tests were conducted to compare sleep-related metrics between weeks 4 and 8 (Table 3). The results showed a significant decrease in the PSQI sleep latency score (β = −0.3, *p* = 0.031), indicating faster sleep onset. Other metrics, such as the PSQI global score (*p* = 0.143) and PSQI sleep quality score (*p* = 0.103) showed trends towards improvement, although they did not reach statistical significance. No significant differences were found in other PSQI sleep components, including sleep duration, sleep disturbance, daytime dysfunction, or the use of sleep medication/supplements between the two-time points. From the linear mixed effect model, the result adjusted for race, gender, age, and ethnicity showed an overall decrease in PSQI global score which indicated better self-reported global sleep quality after the sleep intervention. There were no significant changes in the physiologic data (HRV, REM sleep duration, total sleep duration), nor BDI-II scores before and after the implementation of the intervention. The highest absolute value of effect size was 0.47 from the PSQI sleep latency subcomponent, indicating a moderately strong negative effect. Both the PSQI Global score (−0.33) and PSQI Quality subcomponent (0.35) had an effect size larger than 0.3.

**Table 3.** Preliminary differences in sleep and stress metrics before and after Somni© sleep kit implementation

Characteristic	Mean Difference	SD	*p*	Cohen’s D
**Measure**				
Pittsburgh Sleep Quality Index (PSQI)				
PSQI Global score	−0.57	1.720	0.14	−0.33
Sleep Quality component	0.17	0.482	0.1	0.35
Sleep Latency component	−0.3	0.635	0.03	−0.47
Sleep Duration component	−0.09	0.668	0.54	−0.13
Sleep Efficacy component	−0.14	0.990	0.53	−0.14
Sleep Disturbance component	−2.68E-16	0.617	1	−4.34E-16
Daytime Dysfunction component	−0.13	0.548	0.27	−0.24
Use of Medications component	1.64E-17	0.798	1	2.06E-17
Oura ring Physiologic Data				
Sleep Duration	0.019	0.255	0.71	0.075
REM sleep duration	0.010	0.186	0.78	0.054
Heart Rate Variability	0.846	5.424	0.44	0.16
Beck Depression Inventory-II	−1.4	5.325	0.25	−0.26
Maslach Burnout Inventory				
Emotional Exhaustion	0.833	17.798	0.82	0.047
Depersonalization	−0.042	8.24	0.98	−0.005
Professional Accomplishment	0.292	9.37	0.88	0.0312
Perceived Stress Scale	0.13	2.912	0.8319	0.0446

**Figure 1.**
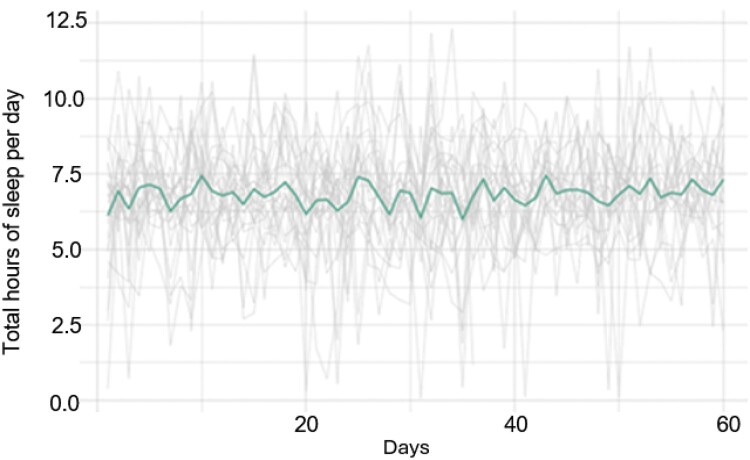
Physiologic daily sleep duration data over 8 weeks among 25 nurses.

**Figure 2.**
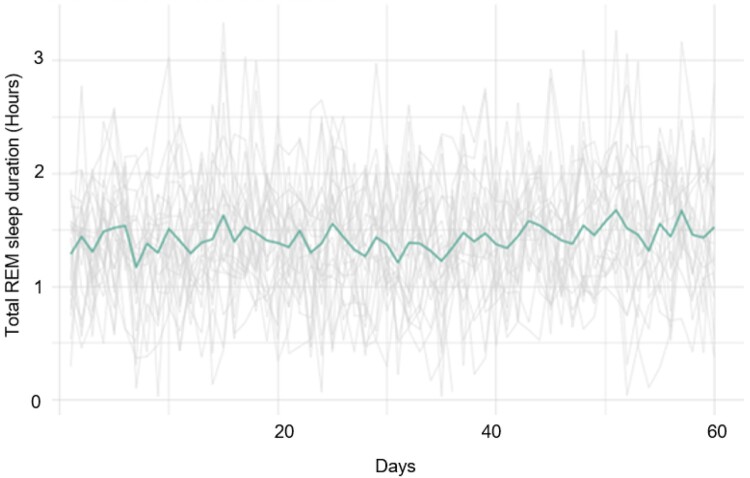
Group-level mean duration of daily sleep hours spent in REM cycle.

Figures 1 and 2 provide a visual snapshot of group level sleep duration and time spent in the REM cycle. On average participants met the recommended 7–8 hours/day for sleep duration. However, there were several individuals that had less than 3 hours of sleep in a 24-hour time period on multiple days. At least 50% of participants did not reach the recommended 2-hour duration of time spent in the REM cycle.

## Discussion

This study investigated the preliminary impact of sleep quality promotion items on nursing workforce sleep, stress responses and mental health. Overall, the survey results suggest that the sleep aids may have varying effects on self-reported sleep quality, and longitudinal data indicates potential improvements in sleep latency over time. It was also evident that there is a variability of individual preference for type of sleep aids. Of those devices provided, aromatherapy and white noise was the most used despite light blocking glasses being perceived as the most influential on sleep quality. The noted variability between the frequency of use and perceived impact should be investigated with larger sample sizes and with more specific control of the use of aids to confirm these findings and explore the long-term effects of different sleep aid interventions in this population.

Over 20% of the sample reported frequent use of pharmacologic or supplemental sleep aids (e.g. Ambient, melatonin) that were not included in the Somni© sleep kit administered in this study. Of these participants, upward of one-third perceived pharmacologic aids as being helpful in improving sleep quality. There was, however, interest in the non-pharmacologic aides that were offered, specifically, lavender spray and to a lesser degree white noise machine, ear plugs and eye masks. Interestingly, participants reported that they perceived Somni© sleep kit items like lavender spray, eye mask, and blue blocking glasses improved their sleep as much as or more than sleep medications. This suggests that non-pharmacologic approaches to improve sleep are appealing to this nursing cohort and their preferences for use and impacts on sleep should be further examined. As aligned with previous research, various interventions – ranging from aromatherapy to shift schedule modifications – show moderate effectiveness in improving nurses’ sleep, but inconsistent outcomes limit definitive conclusions, highlighting the need for more rigorous research (Zhang et al., 2023).

The results of our small pilot study are mixed, showing some effectiveness of the Somni intervention on improving sleep. Specifically, the Somni© sleep kit appeared to be more effective in improving self-reported sleep quality than sleep duration, as assessed via self-report or the Oura ring. This may not be surprising, considering the Somni© sleep kit items are aligned with fostering a relaxing and sleep conducive environment (i.e. are sleep hygiene tools). Other approaches, such as behavioral sleep extension, including specifying sleep scheduling, may be more effective in increasing sleep duration (Zheng et al., 2024). Our results appear consistent with a recent review where the authors, Albakri et al. (2021) reviewed literature to assess which sleep interventions were the most studied and efficient in improving sleep duration and quality. The authors reported that among various types of interventions, there was little evidence that resulted in any one type of intervention being better than others. Some were promising, such as mind–body exercise, physical exercise, aromatherapy and/or massage interventions, behavioral change (mostly in children), while other interventions appeared to have mixed results.

This study supported the initial feasibility and acceptability of nurses using Oura rings to obtain sleep and heart rate data in real-world studies. Evidence about mobile digital devices used by health workforce in real-world clinical settings continues to emerge and involves adaptive changes in human attitude and skills to adopt technology (Socha-Dietrich, 2021). The expansion of research approaches at the crossroads of health services research and precision medicine may direct workforce policies that are best supported by physiologic stress measurement, as opposed to self-report. Future research should continue to explore novel methods and devices that may better capture the impact of interventions promoting clinical workforce health outcomes. Specifically, the data from this pilot study will be employed in a longitudinal cohort study that uses quota sampling to account for shiftwork and setting, while also tailoring device intervention exposure.

There are limitations to this study. As this was a pilot trial, the small sample size did not generate enough power to demonstrate significance, but the data acquired will be used to inform future larger studies. Recruitment of the study sample was limited to a single hospital which threatens generalizability. Further, survey measures used may be impacted by self-report bias. There were no specific sleep inclusion criteria, i.e. enrollment did not target specifically those individuals with poor sleep or insomnia symptoms, or short sleep duration. Since this pilot study recruited a convenience sample, limited by funding for 25 participants, and lacked randomization methods, there is potential for selection bias. Use of the Somni© sleep kit items was based on self-report and could be subject to bias. The study employed a single arm pre-post design, which while allowing an examination of potential intervention effects, would be strengthened in future work by including a control comparator.

## Conclusion

Individualized preferences for sleep quality promotion devices among nurses may improve latency time and perceived quality of sleep. The collection of longitudinal physiologic data of nurses in real-world clinical settings appears feasible and acceptable with use of the Oura ring device. [maybe we can add in a sentence or two about the burden of sleep disturbance in nurses, and its links to psychological distress and burnout. hence the need for more work to develop methods to improve sleep in this population.] Future research investigating sleep quality among nursing workforce should include larger, generalizable samples.

## Data Availability

The data in this study is available upon reasonable request.
